# Removing the Clitoris in Cases of Masturbation, Accompanied with Threatening Insanity

**Published:** 1862-05

**Authors:** E. S. Cooper

**Affiliations:** Professor of Anatomy and Surgery in the Medical Department of the University of the Pacific


### REMOVING THE CLITORIS IN CASES OF MASTURBATION,

### ACCOMPANIED WITH THREATENING INSANITY.

BY E. S. COOPER, A. M., M. D.,

*Professor of Anatomy and Surgery in the Medical Department of the University of the Pacific.*

The proper management of inveterate cases of masturbation, in both sexes, is a subject of great importance to medical men, in view of the fact of the practice causing a degeneration of intellect, and even insanity itself. These case are far more numerous, in every country, than they are supposed to be, except by medical men. Being subjects of great delicacy, they remain, as they should be, secrets between the family and medical adviser. Though these are matters of a very private nature, they should always be freely discussed by medical men the more so, since very little has, thus far, been done to ameliorate the condition of the more unfortunate victims of this practice, who have progressed towards approaching insanity. But to the inexpressible anguish and chagrin of their parents and the most profound pity of all who know anything of them, they often degenerate, physically, morally and intellectually, until they pass out of the reach of medical aid, and become hopelessly lost. At no time does the practitioner feel more keenly the inability of his art to save, however often he may have to deplore his inefficiency in arresting the progress of some fatal malady. Death is infinitely preferable to insanity from this cause.

What can be more deplorable than to see a little girl, twelve or fourteen years old, the daughter of intelligent, high-toned parents, and naturally bright herself, but so far advanced in the practice of masturbation and mental degeneracy as to be entirely unable to govern herself, even after the habit is observed, and the fearful consequences of continuance fully explained to her? Or what can call forth, to a greater extent, our heartfelt commiseration, than to see a youth, with fine mental endowments by nature, just verging into manhood, finding his mental and moral energies failing, under the influence of a practice, the noxiousness of which he had no idea, at the time of commencing it, but who, at last, finds a mental wreck almost inevitable, and who implores the surgeon to save him by any means, even that of castration. Though surgeons would hardly be justified in performing this operation, still in view of the awful results so often growing out of the practice of masturbation, when it has produced incipient insanity, and deprived the patient of the moral courage and firmness necessary to control the habit, we would be justified in resorting to almost any operation.

In the female, it would appear, judging from the result of two cases, as though we have found a remedy by surgical interference, and of such mildness and simplicity as to commend it to an early trial in all cases where the habit of masturbation threatens injury of the intellect. It consists in removing the entire clitoris, including the corpus cavernosum clitoridis, and the major portion of the erectores clitoridis. We will briefly detail the cases:

*Case 1st.*—Miss ———, æt. 13 years and 6 months, once a beautiful bright little girl, whom I knew in the Atlantic States, eight years previously, was brought to me in July, 1860. At first sight of her, it was easy to recognize the bland, childish smile, which was one of her striking characteristics at the time I had seen her last. But, in conversation, I soon found that she had lost her former vivacity and brightness of intellect, but when we began to speak of her condition—which was begun by her mother, whose mortification at having to come upon such an errand, induced her to speak more from feeling than prudence—the little girl's face changed to a sort of idiotic ferocity, which made it but too apparent that insanity was fast absorbing her faculties. Although so young, she had already attempted to commit suicide, by trying to cut her throat with a razor. There appeared little chance of doing anything for her. She took, however, Lupulin in 5-grain doses, three times a day, for some time, and her mother was admonished, in the strongest manner, to change her harsh tone towards her, and to endeavor to win her confidence and secure obedience, by kindness. This course was continued for two months, but with no effect. We could neither improve the condition of the nervous system, nor induce her to leave off her practice. The mother had her watched, day and night, and thought she might, in that way, prevent it altogether. But the child had a mode of crossing her thighs, and rubbing with them at the same time, so as to produce the desired result. This she would sometimes do with so little movement, especially while in bed, as to deceive those who were watching her, even after their attention was directed to the fact; and, what was singular in regard to this, sometimes she would confess, candidly, what she had been doing, and as often deny, most persistently, the practice altogether. The mother, being now almost frantic, had abandoned all hope of saving her from a permanent residence in the Lunatic Asylum, and implored me to do everything, try every experiment that offered the least probability of cure. The removal of the clitoris was then proposed, and its trial most willingly consented to by the mother.

*Operation.*—The operation consists in grasping the clitoris, back of the glans, with a pair of forceps, drawing it forwards, so as to place the erectores clitoridis and corpus spongiosum upon the stretch, when the whole mass was cut away, by the scalpel, with small portions of the nymphæ. Very little hemorrhage occurred, and the patient was quite comfortable the next day, though very melancholy, and recovered, without an untoward symptom.

The operation was successful in breaking up the practice, and the patient has very much improved in the mental faculties, all save the memory, which appears to be almost entirely destroyed.

*Casi* 2.—*Miss*——, œt. 10 years, was discovered to be in the habit of committing Masturbation, about six months prior to my being consulted, June, 1861. She was thought, by her mother, to be gradually becoming stupid, for some months previously, and her nervous system had lost, almost entirely, its wonted tone.

In July following, with the assistance of Prof. L. C. Lane, I removed the cliteris, as before. The patient suffered no particular inconvenience from the operation; remained in bed one day, and walked about, after that, as if nothing had occurred.

The result has been entirely satisfactory. The habit was broken, and the little girl began rapidly to regain her usual sprightliness, and is now from home at a boarding-school, whence she often sends letters to her friends. The letters show her to be a girl of extraordinary intellect, though, prior to the operation she had occasionally a vacant idiotic stare, which promised anything but her present bright state of mind. Her parents informed me that she had taught a little sister of hers, only two years old, the same habit, and that, at one time she thrust one of her fingers up the vagina of the little child. On examining the babe, we found the hyman not only ruptured, but the clitoris and vagina four or six times as much developed as they should be.

*Remarks.*—After these results, I think a surgeon should not only remove the clitoris, in all similar cases, but, if this should prove insufficient to effect a cure, he should remove the entire nymphæ, also, as they are very vascular, and possess erectile tissue of great sensibility, and are said to be parts often titillated in Masturbation.

*Query.*—What effect will these operations have upon the person in a married state? These parts are supposed, by many physiologists, to have much influence over sensual gratification. Some regard them, in fact, as the seat of venereal pleasure.

Practically, this matter does not come up in those desperate cases in which a destruction of the mental faculties is rapidly going on, and there is no other known way of curing the patient. But, in a physiological point of view, it is a subject of great interest.—*San Fransisco Med. Press.*

